# WNT-1 inducible signaling pathway protein-1 enhances growth and tumorigenesis in human breast cancer

**DOI:** 10.1038/srep08686

**Published:** 2015-03-03

**Authors:** Kun-Chun Chiang, Chun-Nan Yeh, Li-Chuan Chung, Tsui-Hsia Feng, Chi-Chin Sun, Miin-Fu Chen, Yi-Yin Jan, Ta-Sen Yeh, Shin-Cheh Chen, Horng-Heng Juang

**Affiliations:** 1 General Surgery Department, Chang Gung Memorial Hospital and Chang Gung University, 222, Mai-Chin Road, Keelung, Taiwan, 204, R.O.C; 2 General Surgery Department, Chang Gung Memorial Hospital and Chang Gung University, 5, Fu-Hsing Street, Kwei-Shan, Taoyuan, Taiwan, 333, R.O.C; 3 Department of Anatomy, School of Medicine, Chang Gung University, 259 Wen-Hwa 1st Road, Kwei-Shan Tao-Yuan, Taiwan, 333, R.O.C; 4 Department of Nursing, School of Medicine, Chang Gung University, 259 Wen-Hwa 1st Road, Kwei-Shan Tao-Yuan, Taiwan, 333, R.O.C; 5 Department of Ophthalmology, Chang Gung Memorial Hospital, 222, Mai-Chin Road, Keelung, Taiwan, 204, R.O.C

## Abstract

WNT1 inducible signaling pathway protein 1 (WISP1) plays a key role in many cellular functions in a highly tissue-specific manner; however the role of WISP1 in breast cancer is still poorly understood. Here, we demonstrate that WISP1 acts as an oncogene in human breast cancer. We demonstrated that human breast cancer tissues had higher WISP1 mRNA expression than normal breast tissues and that treatment of recombinant WISP1 enhanced breast cancer cell proliferation. Further, ectopic expression of WISP1 increased the growth of breast cancer cells *in vitro* and *in vivo*. WISP1 transfection also induced epithelial-mesenchymal-transition (EMT) in MCF-7 cells, leading to higher migration and invasion. During this EMT-inducing process, E-cadherin was repressed and N-cadherin, snail, and β-catenin were upregulated. Filamentous actin (F-actin) remodeling and polarization were also observed after WISP1 transfection into MCF-7 cells. Moreover, forced overexpression of WISP1 blocked the expression of NDRG1, a breast cancer tumor suppressor gene. Our study provides novel evidence that WISP1-modulated NDRG1 gene expression is dependent on a DNA fragment (−128 to +46) located within the human NDRG1 promoter. Thus, we concluded that WISP1 is a human breast cancer oncogene and is a potential therapeutic target.

Breast cancer is the most commonly diagnosed cancer and ranks first on the list of cancer-related death in females^1^. Even with recent improvements in understanding the underlying mechanisms of breast cancer and development of new therapeutic approaches, 226,870 American women are diagnosed with breast cancer and 39,510 women die from this disease annually^2^. Of all cancer patients, breast cancer accounts for 23% of the cases and 14%of cancer-related deaths. Metastasis attributes to almost all breast cancer-related deaths, with approximately 25 to 50% of all breast cancer cases eventually developing metastasis. Once metastasis is diagnosed, the 5 year survival rate is generally less than 25%, even with aggressive treatment^3^,^4^. Thus, to develop new therapeutic strategies to treat metastatic breast cancer, or even to prevent metastasis, should be top priority.

The WNT1 inducible signaling pathway protein 1 (WISP1), located on chromosome 8q24.1–q24.3 and consisting of 5 exons and 4 introns, is a secreted matricellular protein found in the extracellular matrix (ECM). WISP1, like other ECM proteins, affects a variety of cell responses, including differentiation, proliferation, migration, and survival, but is not needed for structural maintenance^5^. The murine WISP1 homologue was the first to be discovered, and is differently expressed in cells with low or high metastatic ability^6^. Human WISP1 was identified in C57MG cells, a human mammary epithelial cell line with WNT-1 expression, and shown to be a WNT-1-induced gene in 1998^7^.

WISP1 has been suggested to affect cell survival and proliferation. For example, WISP1 has a mitogenic effect on rat fibroblasts *in vitro* and *in vivo*^8^. WISP1 overexpression in stromal cells in the proximity of tumors caused an increase in tumor growth via paracrine signaling^9^,^10^. Contrary to its oncogenic role, WISP1 also functions as a tumor suppressor gene. For example, WISP1 transfection into melanoma cells repressed tumor cell growth^6^. Further, overexpression of WISP1 down-regulated the invasion and migration of lung cancer cells, leading to reduced metastatic potential^11^. The finding that WISP1 expression is increased in carcinoma cells *in vivo*, including colon, lung, liver, and breast cancer further confounds the role of WISP1 in carcinogenesis^7^,^12^,^13^,^14^.

N-myc downstream-regulated gene 1 (NDRG1), a protein induced by stress^15^, is a tumor suppressor gene in a variety of cancers^16^,^17^,^18^. For breast cancer, NDRG1 represses breast cancer cell proliferation and invasion when ectopically overexpressed^19^.

Because the role of WISP1 in breast cancer is unclear, in this study, we investigated the role and underlying mechanisms of WISP1 in human breast cancer. Further, we demonstrated for the first time the mechanisms by which WISP1 modulates NDRG1 expression in human breast cancer cells.

## Results

### Identification of WISP1 as an oncogene for human breast cancer

**Figure 1:**
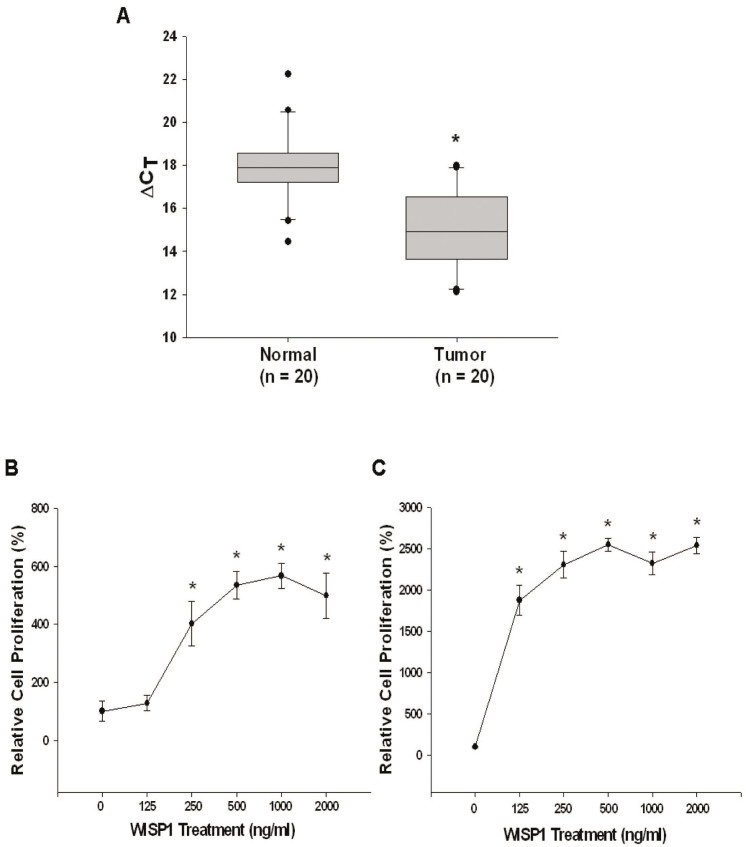
WISP1 plays as on oncogene in human breast cancer. (A) Quantitative analysis of WISP1 expression in breast cancerous and normal tissues by RT-qPCR. Box plots analysis was used to compare the WISP1 expression in cancerous and normal breast tissues. Recombinant WISP1 was used to treat MCF-7 cells (B) or MDA-MB-231 cells (C) a concentration ranging from 0 to 2000 ng/mL for 2 days. Cell proliferation was measured using the CyQUANT cell proliferation assay kit. Each point of the curve represents the mean percentage ± SE (n = 6) in relation to that of control-solvent groups. (*P < 0.01).

RT-qPCR results showed that the mean between 20 pairs of normal and cancerous tissues is ΔΔC_t_ = −2.87 + 0.53, indicating that WISP1 mRNA expression is higher in breast cancer tissues as compared to normal breast tissues (Fig. 1A). To further verity the role of WISP1 in breast cancer, we treated two human breast cancer cell lines, MCF-7 (ER+) and MD-AMB-231 (ER-), with recombinant WISP1 protein. As shown in Fig. 1B and 1C, WISP1 (from 0 to 1000 ng/mL) increased MCF-7 cell proliferation in a dose dependent manner. Cell proliferation also increased in MD-AMB-231 cells treated with recombinant WISP1 (from 0 to 500 ng/mL) in a dose-dependent manner. Thus, based on this result, we concluded that WISP1 functions as an oncogene for human breast cancer.

### Evaluation of WISP1 expression and distribution in MCF-7 cell

**Figure 2:**
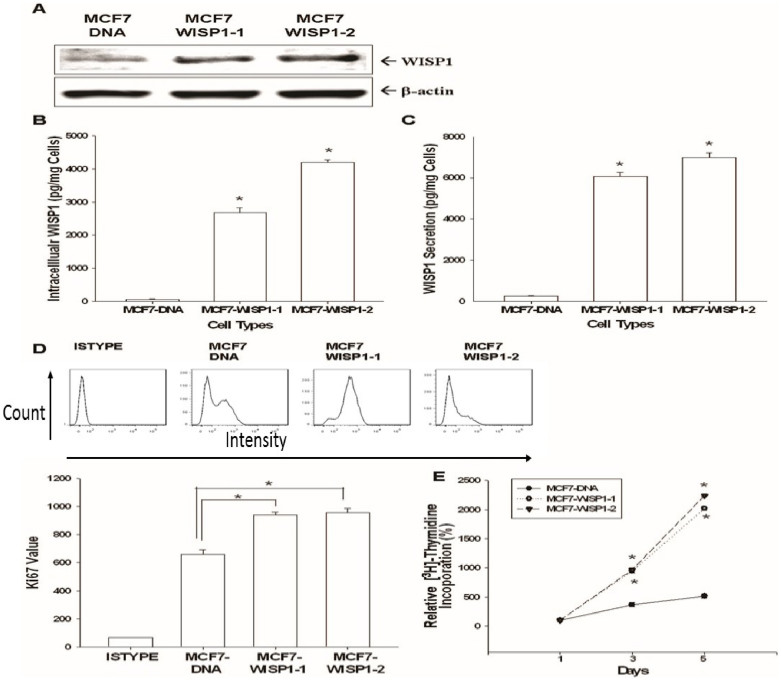
Effect of ectopic WISP1 overexpression on cell proliferation in MCF-7 cells. (A) Western blot depicting WISP1 expression in MCF7-DNA, MCF7-WISP1-1, and MCF7-WISP1-2 cells. The WISP1 levels of intracellular components (B) and condition media (C) of cells were measured by ELISA. Data are presented as the mean ± SE (n = 6). The effect of ectopic WISP1 expression on MCF-7 cell proliferation was determined by Ki67 expression measured by flow cytometry (D). ISYTYPE represents the negative control group. Data are presented as the mean ± SD (n = 3). ^3^H-thymidine incorporation assays (E) were also applied to measure cell proliferation. Data are presented as the mean percentage ± SE (n = 3–6) in relation to mock-transfected (MCF7-DNA) cells. (*P < 0.01).

The strong expression of WISP1 in two stable clones of MCF-7 cells overexpressing WISP1 (MCF7-WISP1-1 and MCF7-WISP1-2) compared to mock-transfected cells (MCF7-DNA) was confirmed by western blot (Fig 2A). Since WISP1 is a secretory protein, intracellular and extracellular WISP1 levels also were measured by ELISA. As shown in Fig 2B and 2C, WISP1 was detected in MCF7-WISP1-1 and MCF7-WISP1-2 cells in both the intracellular and extracellular components at 2800 to 7000 pg/mg cells.

### Evaluation of WISP1's effect on MCF-7 cell growth and the related mechanisms

**Figure 3:**
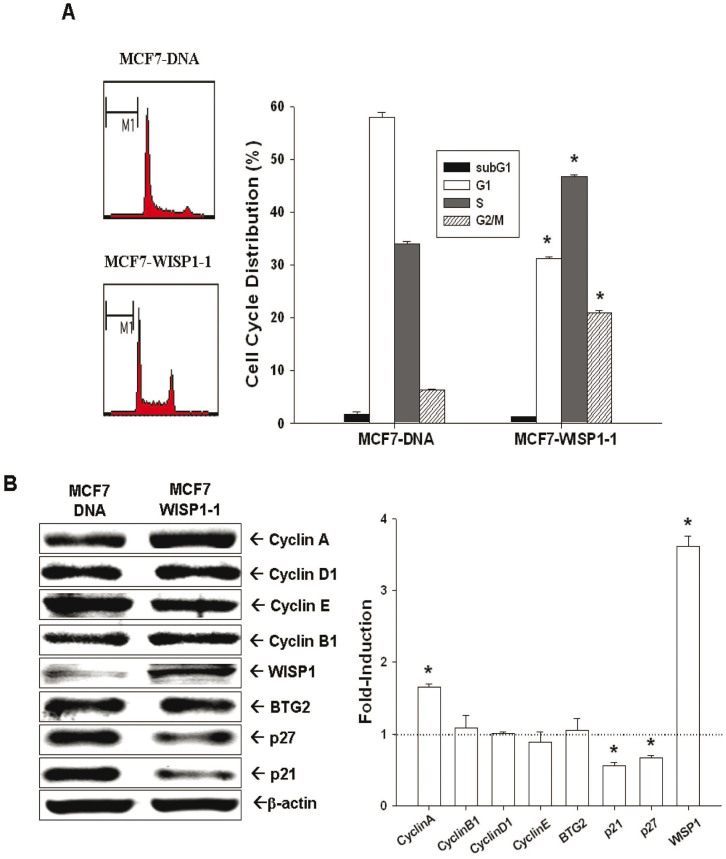
Effect of ectopic WISP1 overexpression on cell cycle distribution of MCF-7 cells. (A). The cell cycle distribution of MCF7-DNA and MCF7-WISP1-1 cells was analyzed by flow cytometry after 48 hours incubation. The data shown in each bar chart represent the mean percentage ± SE (*n* = 5) of cells in each phase of the cell cycle. (B). The expression (Cropped) and quantitative analysis of cyclins, p21, p27, WISP1, and BTG2 in MCF7-DNA and MCF7-WISP1-1 cells were determined by immunoblotting assays. Data are presented as fold-induction of target genes after WISP1-overexpressed. (*P < 0.01).

Ectopic expression of WISP1 significantly increased Ki67 expression (Fig. 2D) and ^3^H-thymide incorporation (Fig. 2E) in MCF-7 cells. The doubling times for MCF7- DNA, MCF7-WISP1-1, and MCF7-WISP1-2 are 48.22, 30.45, and 23.87 hours, respectively, which were determined by two time points of cell viability measured using the WST-1 method. As analyzed by flow cytometry of cell cycle distribution, WISP1 overexpression increased the percentage of cells in S phase from 34 ± 3% to 46 ± 2% (Fig 3A). We also measured the expression of the tumor suppressor gene BTG2, and the expression of several cell cycle related cyclins and cyclin-dependent kinase inhibitors (CKIs). As shown in Fig 3B, MCF7-WISP1-1 cells presented higher levels of cyclin A and lower levels of the CKIs p21 and p27 than MCF7-DNA cells. However, the expression of BTG2, cyclin D1 and cyclin E was not influenced by WISP1 overexpression. Based on this result, we concluded that WISP1 stimulates MCF-7 cell growth by down-regulating p21 and p27, leading to increased S phase cells that present with high cyclin A.

### Evaluation of WISP1's effect on MCF-7 cell metastasis potential and related mechanisms

**Figure 4:**
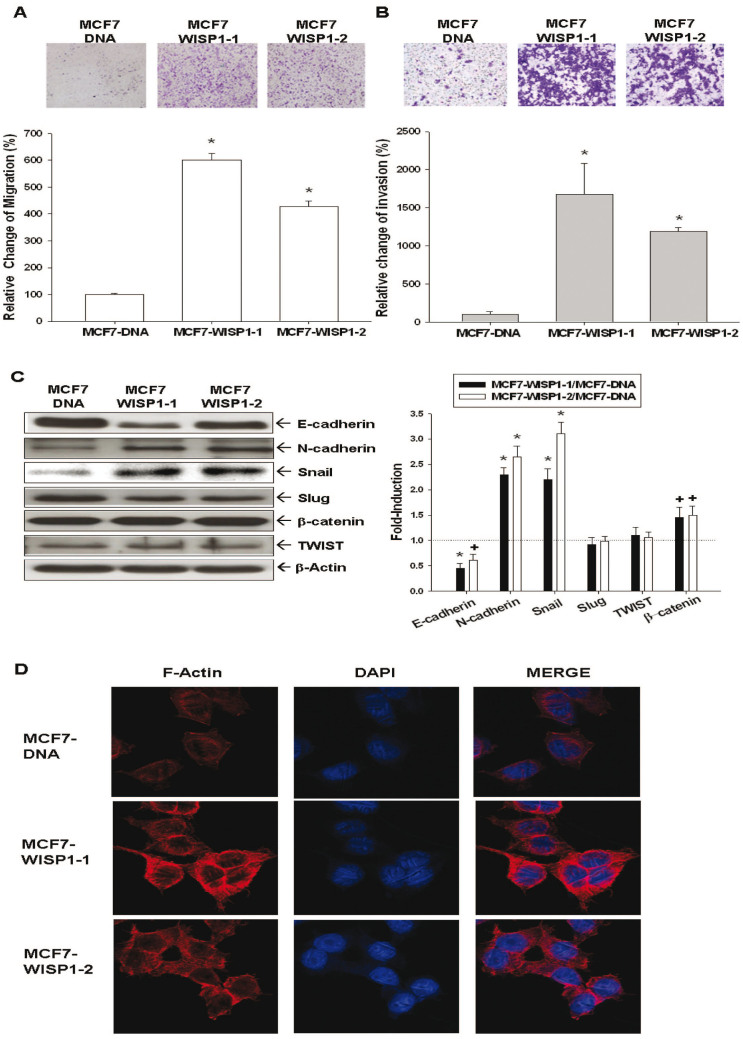
Effect of ectopic overexpression of WISP1 on cell migration, cell invasion, epithelial-mesenchymal transition markers, and F-actin polarization of MCF-7 cells. The cell migration (A) and invasion (B) of MCF7-DNA, MCF7-WISP1-1, and MCF7-WISP1-2 cells was determined by trans-well filter without and with Matrigel-coated membranes. The migrating or invading cells were digitally photographed and then counted under the microscope. Experiments were performed in triplicate and repeated at least three times, and the data of quantitative analysis were expressed as average cell counts/9 fields ± SE (*P < 0.01). (C) Gene expression of epithelial-mesenchymal transition markers in MCF7-DNA, MCF7-WISP1-1, and MCF7-WISP1-2 cells was determined by western blot assays (Cropped). The fold-induction data are expressed as the intensity of the protein bands produced by the target gene/β-actin (± SE; *n* = 3) relative to that of the MCF7-DNA cells (*P < 0.01; ^+^*P* < 0.05). (D) Immunofluorescence staining of F-actin (red) expression and distribution of MCF7-DNA, MCF7-WISP1-1, and MCF7-WISP1-2 cells. DAPI (blue) was applied for nuclear staining.

The migration (Fig. 4A) and invasion (Fig. 4B) abilities, as determined by trans-well filter without and with Matrigel-coated membranes, respectively, were significantly higher in MCF7-WISP1-1 and MCF7-WISP1-2 cells than in MCF7-DNA cells, Since epithelial-mesenchymal-transition (EMT) plays a crucial role during cancer metastasis, EMT-related proteins were then investigated. As shown in Fig 4C, both MCF7-WISP1-1 and MCF7-WISP1-2 cells expressed lower levels of E-cadherin and higher levels of N-cadherin, snail, and β-catenin, while the expression of slug and twist was unaffected.

### Evaluation of WISP1's effect on F-actin synthesis and polarization in MCF-7 cells

As shown in Fig 4D, cells were double stained with anti F-actin antibody (red) and DAPI (green) for nuclear staining, and immunofluorescence intensity and distribution were observed using confocal microscopy. F-actin expression within the cytoplasm and F-actin polar distribution were more prominent in MCF7-WISP1-1 and MCF7-WISP1-2 cells than in MCF7-DNA cells, indicating that WISP1 overexpression increased F-actin synthesis and polarization in MCF-7 cells.

### Evaluation of WISP1's effect on NDRG1 expression in MCF-7 cells

**Figure 5:**
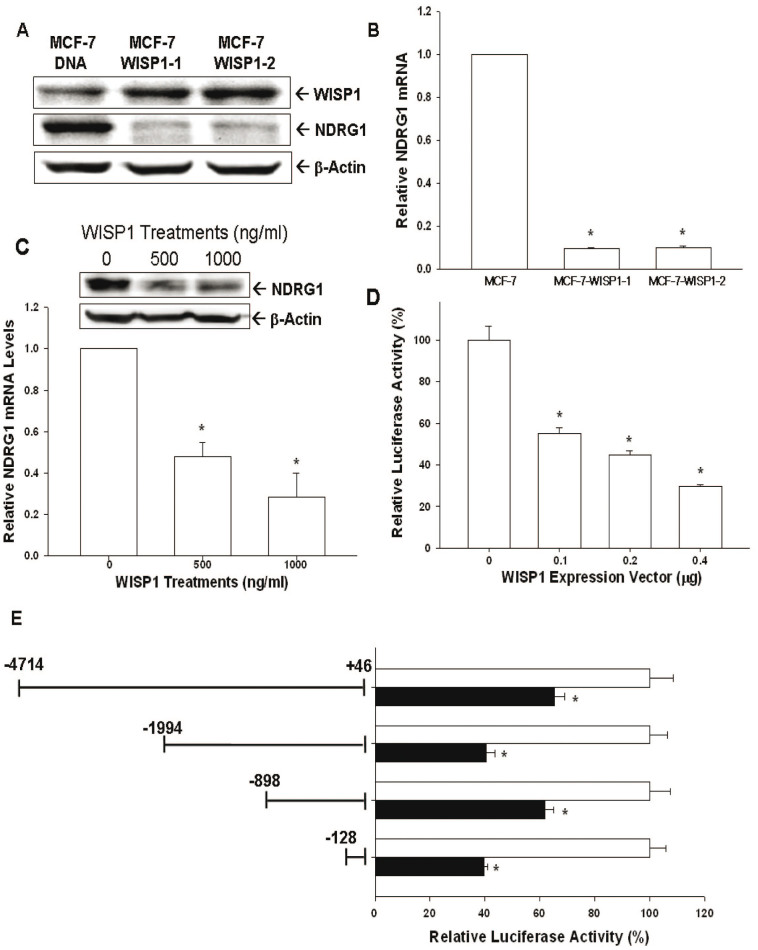
Identification of NDRG1 as the downstream of WISP1 in MCF-7 cells. NDRG1 expression in MCF7-DNA, MCF7-WISP1-1, and MCF7-WISP1-2 cells was determined by western blot (A) and RT-qPCR (B). NDRG1 expression of MCF-7 cells after treatment with recombinant human WISP1 protein as determined by western blot (top) and RT-qPCR (bottom) (C). (D) The NDRG1 reporter vector containing the human NDRG1 promoter/enhancer DNA fragment (−4714 to +46) was co-transfected with different concentrations of WISP-1 expression vector into MCDF-7 cells. The luciferase activity of the NDRG1 reporter in MCF-7 cells was presented as the mean percentage ± SE (n = 6) in relation to no WISP-1 expression vector transfection group. (E) Relative luciferase activity of reporter vectors containing different fragments from the NDRG1 promoter/enhancer as indicated. Data are presented as mean percentage ± SE (n = 6) of the luciferase activity in relation to mock-transfected cells (*P < 0.01).

**Figure 6:**
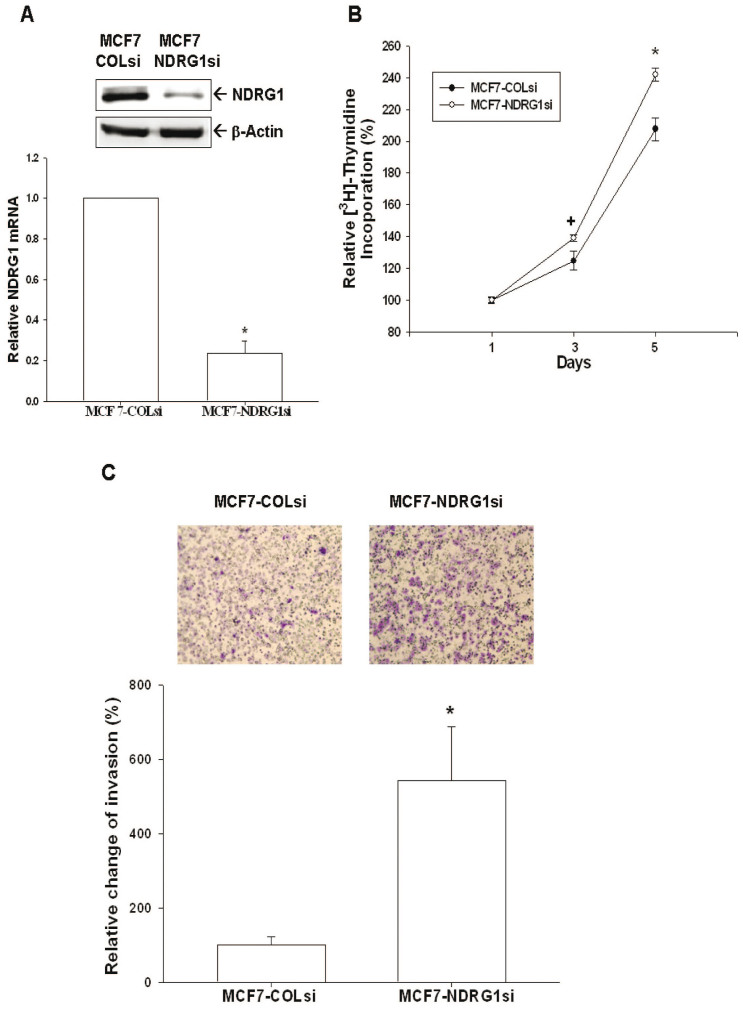
Knockdown NDRG1 enhances cell proliferation and invasion of MCF-7 cells. (A) The expression of NDRG1 in NDRG1-knockdown MCF-7 (MCF7-NDRG1si) cells and mock-knockdown MCF-7 (MCF7-COLsi) cells were determined by immunoblotting (top) and RT-qPCR (bottom). Data are expressed as mean ± SE (*n* = 3) and the NDRG1 level of MCF-COLsi cells is set as 1(*P < 0.01). (B) Cell proliferation of MCF7-COLsi (•) and MCF7-NDRG1si (○) cells was determined by the^3^H-thymidine incorporation assay. Each point on the curve represents the mean percentage ± SE (*n* = 4) in relation to Day 1. (C) The cell invasion ability of MCF7-COLsi and MCF7-NDRG1si cells after 48 hours incubation was measured using the matrigel-invasion assay. Experiments were performed in triplicate and repeated at least three times. Data of quantitative analysis are expressed as average cell counts/9 fields ± SE of MCF7-COLsi and MCF7-NDRG1si cells. (*P < 0.01).

Western blot (Fig. 5A) and RT-qPCR (Fig. 5B) suggested that WISP1 represses NDRG1 expression in MCF-7 cells, as indicated by the reduced expression of NDRG1 in MCF7-WISP1-1 and MCF7-WISP1-2 cells compared to MCF7-DNA cells. Treating MCF-7 cells with different concentrations of WISP1 recombinant protein caused NDRG1 expression to decrease significantly, as determined by western blot and RT-qPCR (Fig. 5C). As we treated MCF-7 cells with different concentrations of WISP1 expression vectors, the NDRG1 reporter assay in MCF-7 cells showed a dose-dependent activity downregulation (Fig 5D). The 5′-deletion NDRG1 reporter assay further confirmed that WISP1 response element is located within the promoter area (−128 to +46) of NDRG1 gene (Fig 5E). To further verify the role of NDRG1 in MCF-7 cells, we knocked down NDRG1 by shRNA (Fig 6A) and showed that MCF7-NDRG1si cells exhibited more proliferative and invasive capabilities than MCF7-COLsi cells (Fig. 6B and 6C).

### Evaluation of WISP1 effect on MCF-7 cell growth in vivo

**Figure 7:**
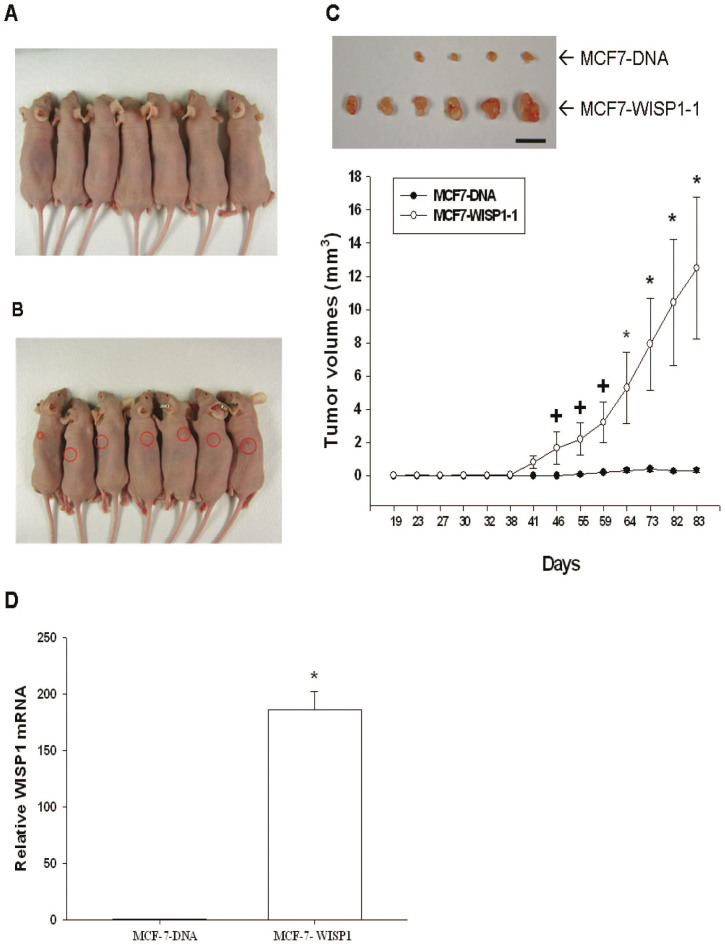
Evaluation of the effect of WISP1 overexpression on MCF-7 cell tumorigenesis and progression *in vivo*. MCF7-DNA (A) and MCF7-WISP1-1 (B) cells (5 × 10^6^) were equally mixed with matrigel and then injected subcutaneously into the back area of each nude mouse. The tumor volumes (mm^3^) in the MCF7-DNA group (•) and the MCF7-WISP1-1 group (○) were measured regularly (C). Data are presented as the mean ± SE. Scale bar, 10 mm. (D) Whole-cell lysates of randomly selected tumor samples from MCF7-DNA (A) and MCF7-WISP1-1 groups were subjected to RT-qPCR. Data are presented as mean fold induction of the WISP1 mRNA (± SE; *n* = 3) relative to the mock-transfected xenograft groups. (^+^*P* < 0.05, *P < 0.01).

After inoculation of 5 × 10^6^ MCF7-DNA cells or MCF7-WISP1-1 cells into the backs of nude mice, the xenografted tumor in MCF-7-DNA group was barely visible during the experimental period (Fig. 7A). Tumorigenesis was first detected in the MCF7-WISP1-1 group 41 days after inoculation, and tumor volume increased steadily until the end of the study (day 83), with all six mice bearing with the tumor (Fig. 7B and 7C). At the end of the study, small tumors were extracted from four out of six mice in the MCF7-DNA group (Fig. 7C). WISP1 mRNA expression was much higher in xenografted MCF7-WISP1-1 tumors than in xenografted MCF-7-DNA tumors, as determined by RT-qPCR (Fig. 7D).

### NDRG1-overexpressed attenuates the effect of WISP1 on cell proliferation and invasion

**Figure 8:**
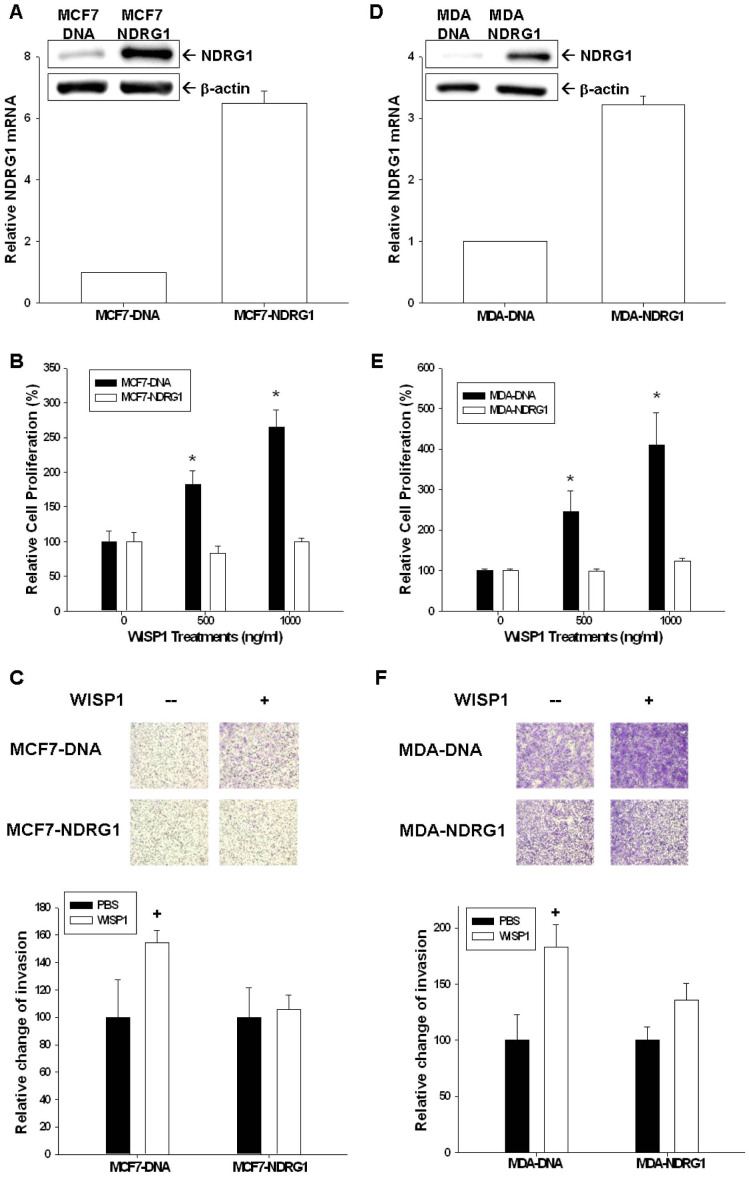
NDRG1-overexpression mitigates the effect of WISP1 in breast cancer cell proliferation and invasion. NDRG1 expression in transiently NDRG1-transfected MCF7 (MCF7-NDRG1) (A) and MDA-MB-231 (MDA-NDRG1) (D) cells was determined by western blot and RT-qPCR. Recombinant WISP1 was used to treat MCF-7 and MCF7-NDRG1 cells (B) or MDA-MB-231 and MDA-NDRG1cells (E) with the indicated concentrations for 2 days. Cell proliferation was measured using the CyQUANT cell proliferation assay kit. The cell invasion ability of MCF-7 and MCF7-NDRG1 cells (C) or MDA-MB-231and MDA-NDRG1 cells (F) after 48 hours incubation with 1000 ng/ml of recombinant WISP was measured using the matrigel-invasion assay. Each point of the curve represents the mean percentage ± SE (n = 6) in relation to that of control-solvent cells. (^+^*P* < 0.05, *P < 0.01).

We transiently overexpressed NDRG1 in MCF-7 (MCF7-NDRG1) and MDA-MB-231 (MDA-NDRG1) cells, as confirmed by western blot and RT-qPCR (Fig 8A and 8D). The ability of recombinant WISP1 protein to activate cell proliferation (Fig. 8B and 8E) and invasion (Fig. 8C and 8F) was significantly attenuated in MCF7-NDRG1 and MDA-NDRG1 cells when compared to MCF7-DNA and MDA-DNA cells, respectively, as determined by CyQUANT cell proliferation assay and by trans-well filter with Matrigel-coated membranes.

### WISP1 is a downstream target of WNT1 in MCF-7 cells

WISP1 is known to be a target of WNT1 in a variety of cells. To verify this relation in MCF-7 cells, we transfected WNT1 into MCF-7 cells. As expected, overexpression of WNT-1 increased WISP1 expression as determined by western blot (Fig. Suppl. 1A) and RT-qPCR (Fig. Suppl. 1B).

## Discussion

WISP1 is a member of the CCN protein family which originally included the proteins cysteine rich 61 (CYR61/CCN1), connective tissue growth factor (CTGF/CCN2), and nephroblastoma overexpressed (NOV/CCN3). In 1998, Pennica *et al*. identified three additional WISP proteins, including, WISP1, WISP2, and WISP3, which later were assigned to the CCN family and renamed as CCN4/WISP1, CCN5/WISP2 and CCN6/WISP3^20^. Among the CCN protein family, CCN2 previously was shown to increase migration and angiogenesis in breast cancer cells^21^. WISP1 (CCN4) is mainly expressed during organ development and under diseased conditions, such as fibrosis or cancer^22^. However, the exact function of WISP1 still is not well understood and it appears that WISP1 works in a highly cell-specific manner, particularly during cancer development and progression. For example, WISP1 promotes prostate cancer cell growth and metastasis to bone^23^, and also serves as a tumor suppressor gene in melanoma and lung cancer cells^6^,^11^. In terms of breast cancer, the role of WISP1 is still controversial. Davis *et al*. previously showed that WISP1 is likely a tumor suppressor gene for breast cancer^24^; while others have reported that WISP1 expression is associated with more advanced features, including cancer stage, tumor size, lymph node metastasis, and HER-2/neu overexpression^14^. To date, the clear function of WISP1 in human breast cancer has not been determined. Our current results strongly suggest that WISP1 is an oncogene in human breast cancer. As shown in Figure 1A, human breast cancer tissues expressed higher levels of WISP1 mRNA as than normal breast tissues. This result, together with our finding that recombinant WISP1 promoted the growth of MCF-7 and MDA-MB-231 (Fig 1B and 1C) cells, provides the first laboratory evidence, in addition to the human breast cancer specimen data, suggesting that WISP1 acts as an oncogene in human breast cancer. This conclusion is further supported by our finding that MCF7-WISP-1 and MCF7-WISP -2 cells have shorter cell doubling times and show high Ki67 expression and ^3^H-thymide incorporation (Fig 2D and 2E). As shown in Fig 2B and 2C, and in line with previous finding that WISP1 is a secretory protein, WISP1 expression was detected in the intra- and extra- cellular components of MCF-7 cells, with the latter having a much higher concentration.

During cell cycle progression, the coordinated interaction between cyclins and cyclin-dependent kinases plays a crucial role during cell cycle phase transition. Cyclins and cyclin-dependent kinase complexes are inhibited by CKIs, leading to cell cycle arrest. As shown in Fig 3B, the two main CKIs responsible for the G1/S transition, p21 and p27, were repressed by WISP1, resulting in increased cell proliferation, increased S phase cell population (Fig 3A), and increased expression of cyclin A, a gene expressed in S phase (Fig 3B)^9^,^25^. In contrast, expression of cyclin B1, cyclin D1, and cyclin E in MCF-7 cells was not influenced by ectopic WISP1 expression (Fig 3B). Of note, BTG2 expression, a tumor suppressor gene for breast, stomach, bladder and prostate cancers^26^,^27^,^28^,^29^, was not affected by WISP1 overexpression (Fig 3B). Based on these collective results, we concluded that WISP1 increased MCF-7 cell proliferation partly through the downregulation of p21 and p27.

Breast cancer metastasis is the main cause of breast cancer-related death. The tumor migration and invasion of breast cancer cells are necessary steps before metastasis. Our results indicated that ectopic expression of WISP1in MCF-7 cells enhanced tumor migration and invasion (Fig 4A and 4B). E-cadherin, an adhesion protein that regulates cell-cell adhesion, is negatively associated with cancer prognosis^30^. Its loss may induce cytoskeleton deregulation, which increases cell motility and cancer cell invasiveness^30^. On the other hand, aberrant expression of N-cadherin stimulates migration and invasion in cancer cells^31^. As shown in Fig 4C, ectopic expression of WISP1 decreased E-cadherin expression and increased N-cadherin expression, leading to more invasiveness of MCF7- WISP1-1 and MCF7- WISP1-2 cells. EMT, another key factor during cancer metastasis, enhances cancer cell mobility to facilitate metastasis, increases resistance to immune surveillance and chemotherapy, and stimulates other cancer stem cell-like characteristics^32^,^33^,^34^. EMT also is highly implicated in breast cancer metastasis^35^. At present, at least three families of transcription factors are known to be associated with promoting EMT, including the Snail/Slug, ZEB1/2, and Twist families^36^. As shown in Fig 4C, MCF7-WISP1-1 and MCF7-WISP1-2 cells exhibited higher expression of snail than MCF7-DNA cells, while the expression of slug and twist was not significantly different. The snail-downstream gene, β-catenin^33^, was upregulated in MCF7-WISP1-1 and MCF7-WISP1-2 cells. Since F-actin synthesis and polarization play a crucial role in cell migration^37^, we examined F-actin expression and distribution in MCF7-DNA cells, MCF7-WISP1-1 cells and MCF7-WISP1-2 cells using immunofluorescent staining. As shown in Fig 4D, overexpression of WISP1 induced F-actin synthesis and polarization in MCF-7 cells, leading to enhanced migration of cancer cells. Collectively, our results suggested that WISP1 enhanced MCF-7 metastasis potential through downregulation of E-cadherin, upregulation of N-cadherin, induction of EMT, and stimulation of F-actin synthesis and polarization.

NDRG1, belonging to the α/β hydrolase superfamily, is a 43 kD protein consisting of 394 amino acids. In general, NDRG1 is detected ubiquitously, mainly in the cytoplasm, in a variety of tissues responding to stress signal^15^. NDRG1 represses tumor progression and metastasis in a number of cancers and, thus, is deemed as a tumor suppressor gene^16^,^17^,^18^. Myc oncoproteins, including N-Myc and C-Myc, act upstream of NDRG1 to repress NDRG1 expression^38^. In contrast, p53, PTEN, HIF-1α, and vitamin D all have been shown to upregulate NDRG1 expression^18^,^39^,^40^,^41^. In breast cancer cells, forced overexpression of NDRG1 has been demonstrated to repress cell proliferation and invasion^19^. In addition, NDRG1 expression has been linked with enhanced differentiation of breast cancer cells and improved prognosis for breast cancer^42^,^43^. As shown in Fig 6A and 6B, NDRG1 knock down using shRNA increased MCF-7 cell proliferation and invasion, indicating that NDRG1 functions as a tumor suppressor gene for breast cancer, as previously reported. Further, we found that overexpression of WISP1 or treatment with recombinant WISP1 decreased NDRG1 expression in MCF-7 cells (Fig 5A, B, and C). We also detected a dose dependent inhibition of NDRG1 reporter activity in MCF-7 cells expressing WISP1 (Fig 5D). The ability of WISP1 to affect NDRG1 reporter activity was dependent on a response element located at −128 to +46 in the 5′-flanking region of the human NDRG1 gene (Fig 5E). Moreover, overexpression of NDRG1 attenuated the WISP1-induced increase in proliferation and invasion that was seen in MCF-7 and MDA-MB-231 cells (Figure 8). Taken together, our results suggest that WISP1 inhibits breast cancer proliferation and invasion partly through the downregulation of NDRG1 expression, and that WISP1 regulates NDRG1 expression through DNA sequences located in the NDRG1 promoter.

To verify WISP1 function *in vivo*, we xenografted MCF7-DNA cells and MCF7-WISP1-1 cells into nude mice. As shown in Fig 7, MCF7-WISP1-1 cells induced higher tumorigenesis (all mice were found to bear tumors) and tumor progression than MCF7-DNA cells *in vivo*. The xenografted MCF7-WISP1-1 cells also expressed higher levels of WISP1 mRNA (Fig 7D). Taken together with the *in vitro* results, these data strongly suggest that WISP1 acts as an oncogene for human breast cancer.

## Conclusion

Based on our data, WISP1 functions as an oncogene for human breast cancer. Ectopic expression of WISP1 in breast cancer cells promotes cell growth and metastasis, inhibits p21 and p27 expression, and stimulates EMT. NDRG1, a tumor suppressor gene for breast cancer, is a target of WISP1 and is repressed by WISP1 through DNA sequences within the NDRG1 promoter.

## Methods

### Cell culture

Cell lines were purchased and maintained as previously described^44^.

### Expression Vector Constructs and Stable Transfection

The human WISP1 and WNT1 expression vectors were constructed by cloning the WISP1 cDNA (MGC: 103937; Invitrogen, Carlsbad, CA) and WNT1 cDNA (MGC: 103919, Invitrogen), respectively, into the pcDNA3.1/Zeo expression vector (Invitrogen). Proper ligation was confirmed by extensive restriction mapping and sequencing. Electroporation was performed using the ECM 830 (BTX, San Diego, CA) with a single 70 ms pulse of 180 V, and then cells were maintained in RPMI medium with 10% FCS and 100 μg/mL Zeocin (Invitrogen) as previously described^45^. Transformed colonies were examined to evaluate specific gene expression by immunoblotting and RT-qPCR assays.

### Transient NDRG1 overexpression

The human NDRG1 expression vector was constructed as previously described^46^. Electroporation was performed as described above. Mock-transfected MCF-7 (MCF7-DNA) and MDA-MB-231 (MDA-DNA) cells were transfected with the control vector, pcDNA3, in the same manner as NDRG1 transfected cells.

### Knockdown of NDRG1

MCF7 cells were transduced either with NDRG1 small hairpin RNA lentiviral particles (Sc-36021-V; Santa Cruz Biotechnology) (MCF7-NDRG1si) or with control small hairpin RNA lentiviral particles (Sc-10808-V, Santa Cruz Biotechnology) (MCF7-COLsi) as described by the manufacturer. Two days after transduction, the cells were selected with 5 μg/mL puromycin dihydrochloride for five subsequent generations.

### Cell proliferation and Ki-67 expression

The proliferation of MCF7 and MDA-MB-231 cells after treated with recombinant human WISP1 protein (R&D; Minneapolis, MN) was measured using a CyQUANT cell proliferation assay kit as described by manufacturer (Invitrogen). Cell proliferation of MCF-7 cells after mock-transfected or WISP1 overexpression was measured using^3^H-thymidine incorporation assay as previously described^47^. Ki-67 expression was measured as previously described^48^. Each sample was tested in quadruplicate.

### WISP-1 enzyme-linked immunosorbent assay

Cells were incubated in 1 mL RPMI medium as indicated in a 24–well plate (2 × 10^5^ cell/per well). Following incubation, the supernatants from each well were collected for WISP-1 assays. Cell pellets were washed twice with ice-cold PBS and then dissolved in 200 μL PBS. After sonication for 10 seconds, cell extracts were centrifuged at 12000 rpm for 20 minutes. WISP-1 levels in 20 μL cell supernatant or conditional media were measured by WISP-1 enzyme linked immunosorbent assay (ELISA), as described by manufacturer (DY1627; R&D). The WISP-1 level in each sample was adjusted by the concentration of protein in the whole cell extract, which was measured using BCA protein assay.

### Western blot

The detailed procedure was previously described^44^. The antibodies used in the experiment are listed in supplemental Table 1.

### Flow cytometry for cell cycle analysis

Cells were serum starved for 24 hours and then cultured in RPMI 1640 medium with 10% FCS for another 48 hours. The cells were trypsinized, fixed in ethanol, digested with Triton X-100 and ribonuclease, and stained with propidium iodide as previously described^47^. Cell cycle analysis was performed using FACS-Calibur cytometer and CellQuestPro software (BD Biosciences, San Jose, CA); the data were analyzed using ModFit LT Mac 3.0 software.

### Reverse Transcription Real-Time-Polymerase Chain Reaction

Total RNA was isolated from cells using Trizol reagent, cDNA was synthesized, and real-time polymerase chain reaction (qPCR) was performed as described before^47^. FAM dye-labeled TaqMan MGB probes and PCR primers for human WISP1 (Hs00180245_m1) and NDRG1 (Hs00608387_m1) were purchased from Applied Biosystems. For the internal positive control, 18S (Hs03003631_g1), for tissues, or glyceraldehyde-3-phosphate dehydrogenase (GAPDH; Hs99999905_m1), for cells, was used with a FAM reporter dye-labeled TaqMan MGB probe. The analysis of human breast cancer tissues was approved by Chang Gung Memorial Hospital (approval number: 102-2531B).

### F-actin staining

The cells were seeded onto glass bottom culture dishes (MatTek, Ashland, MD) pre-coated with 50 μL fibronectin and allowed to attach overnight. Cells were fixed with 3.7% formaldehyde, permeabilized with 0.1% Triton X-100, and blocked in 1% BSA at room temperature. F-actin protein expression was detected by incubation with Texas Red X-Phalloidin, and immunofluorescence was examined using confocal microscopy (LSM510 Meta, Zeiss, Oberkochen, Germany) as previously described^45^.

### Invasion and migration assay

The migration and Matrigel invasion assay was performed as previously described^46^. Images were captured using a digital camera connected to an inverted microscope (IX71, Olympus, Tokyo, Japan) with PAX-it Digital Image Management & Image Analysis software, and standardized for light intensity. For all staining, nine total fields per slide were analyzed at a magnification of 4X. Each field of vision was counted to evaluate the invasion and metastasis of tumor cells *in vitro* and these numbers were compared among groups. Individual experiments were done in duplicate and repeated three times.

### Reporter assay

The NDRG1 reporter vectors were previously described^46^. The cells were plated onto 24-well plates at a concentration of 1 × 10^4^ cells/well one day prior to transfection. Cells were transiently transfected using the TransFast transfection reagent (0.6 μg/well, Promega Biosciences) with 1 μg/well of reporter vector and 0.5 μg/well of the pCMVSPORTβgal (Invitrogen). The luciferase activity was adjusted for transfection efficiency using the normalization control plasmid pCMVSPORTβgal as previously described^49^.

### Tumor Xenografts

Tumor xenograft studies were performed in accordance with the Guide for Laboratory Animal Facilities and Care as promulgated by Council of Agriculture Executive Yuan, Taiwan. The protocol was approved by the Chang Gung University Animal Research Committee (Permit Number: CGU13-055). Female nude mice (BALB/cAnN-Foxn1, 4 weeks old) were anesthetized intraperitoneally with by injecting each animal with 100 μL of a mixture of 2.5% tribomoethanol and 2.5% tert-amyl alcohol in Tris buffer solution. Equal volumes of MCF7-DNA or MCF7-WISP1-1 cells and Matrigel were combined to enhance the tumorigenic activity of the cells, before a single anterior-dorsolateral subcutaneous inoculation, consisting of 100 μL of the mixture containing 5 × 10^6^ cells, was administered to each mouse. All animal surgeries were performed under anesthesia, and all efforts were made to minimize suffering. Growth of the xenografts was measured using vernier calipers at 7-day intervals. Tumor volume was calculated as π/6 × larger diameter × (smaller diameter)^2^ as described previously^46^.

### Statistical Analysis

The data from each group were analyzed and compared using the student *t*-test. *P*-value < 0.05 was considered as a significant difference. Tumor volumes in experimental animals and controls were calculated using the Mann-Whitney U test. Excel 2007 SPSS statistical software for Windows (SPSS version 10.0, Chicago, IL, USA) was employed to conduct the statistics.

## Author Contributions

K.C.C., C.N.Y. and H.H.J. wrote the manuscript and designed this experiment, L.C.C., T.H.F., C.C.S., M.F.C., Y.Y.J. helped conduct the experiment, S.C.C., T.S.Y. and H.H.J. are in charge of the whole experiment conduction and paper writing.

## Supplementary Material

Supplementary Information supplemental data
