# Supplementary material for: WNT-1 inducible signaling pathway protein-1 enhances growth and tumorigenesis in human breast cancer
Source: Sci Rep. 2015 Mar 3;5:8686. doi: 10.1038/srep08686 (PMC4346832; doi:10.1038/srep08686)
Supplement: Supplementary Information — supplemental data [file srep08686-s1.pdf]

WNT-1 inducible signaling pathway protein 1 enhances growth and tumorigenesis in human breast cancer

Kun-Chun Chiang[1,#], Chun-Nan Yeh[2#], Li-Chuan Chung[3], Tsui-Hsia Feng[4], Chi-Chin Sun[5], Miin-Fu Chen[2], Yi-Yin Jan[2], Ta-Sen Yeh[2*], Shin-Cheh Chen[2*], Horng-Heng Juang[3*]

[1]General Surgery Department, Chang Gung Memorial Hospital and Chang Gung University, 222, Mai-Chin Road, Keelung, Taiwan, 204, R.O.C

[2]General Surgery Department, Chang Gung Memorial Hospital and Chang Gung University, 5, Fu-Hsing Street, Kwei-Shan, Taoyuan , Taiwan, 333, R.O.C

[3]Department of Anatomy, School of Medicine, Chang Gung University, 259 Wen-Hwa 1st Road, Kwei-Shan Tao-Yuan, Taiwan, 333, R.O.C.

[4]Department of Nursing, School of Medicine, Chang Gung University, 259 Wen-Hwa 1st Road, Kwei-Shan Tao-Yuan, Taiwan, 333, R.O.C.

[5]Department of Ophthalmology, Chang Gung Memorial Hospital, 222, Mai-Chin Road, Keelung, Taiwan, 204, R.O.C

# co-first authors

* co-corresponding authors

Supplemental Figure 1

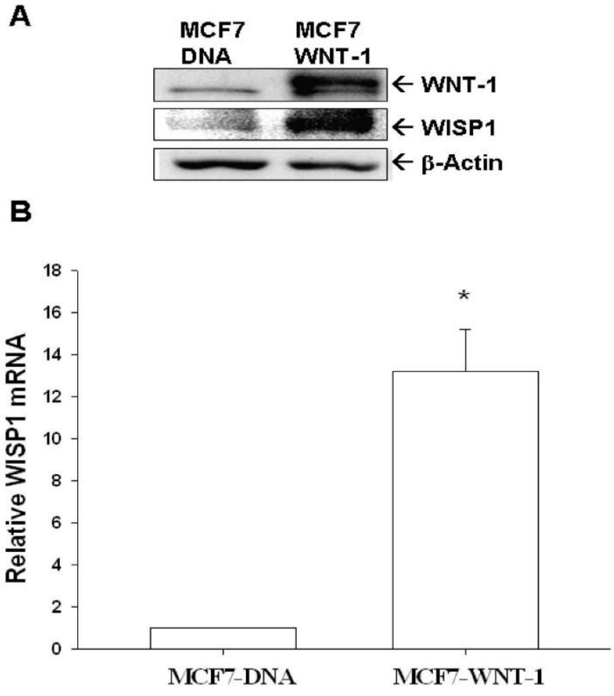

Figure Suppl. 1. WISP1 is WNT1 downstream genes in MCF-7 cells. Expressions of WISP1 and WNT-1 in MCF-7 cells stably transfected with pcDNA3zeoWNT-1 (MCF7-WNT-1) or pcDNA3.1zeo (MCF7-DNA) expression vectors were determined by immunoblotting (A) or RT-qPCR (B).

Supplemental Table: Antibodies information used in this experiment

Antibodies	Brand
Cyclin A	C-19, Santa Cruz Biotechnology
Cyclin B1	D-11, Santa Cruz Biotechnology
Cyclin E	13A3, Santa Cruz Biotechnology
Cyclin D1	DCS6, Cell Signaling, MA, USA
WISP1	SC-8864, Santa Cruz Biotechnology
BTG2	Tsui et al., 2008*
P21	DCS60, Cell Signaling
P27	SX53G8.5, Cell Signaling
NDRG1	42-6200; Invitrogen
E-cadherin	ab53033, Abcam, Cambridge, MA
N-cadherin	AJ1526a , Abgent, San Diego, CA
Snail	sc-28199,Santa Cruz Biotechnology, Santa Cruz, CA
Slug	sc-10436,Santa Cruz Biotechnology, Santa Cruz, CA
Twist	sc-15393,Santa Cruz Biotechnology, Santa Cruz, CA
β-catenin	ab19022,Millipore, Billerica, MA

*Tsui KH, Hsieh WC, Lin MH, Chang PL, Juang HH. Triiodothyronine Modulates cell proliferation of human prostatic carcinoma cells by downregulation of the b-cell translocation gene 2. 2008; The Prostate 68: 610-619.
